# SARS-CoV-2 Nsp5 Activates NF-κB Pathway by Upregulating SUMOylation of MAVS

**DOI:** 10.3389/fimmu.2021.750969

**Published:** 2021-11-10

**Authors:** Weiling Li, Jialu Qiao, Qiang You, Shan Zong, Qian Peng, Yuchen Liu, Song Hu, Wei Liu, Shufen Li, Xiji Shu, Binlian Sun

**Affiliations:** ^1^ Wuhan Institute of Biomedical Sciences, School of Medicine, Jianghan University, Wuhan, China; ^2^ State Key Laboratory of Virology, Wuhan Institute of Virology, Center for Biosafety Mega-Science, Chinese Academy of Sciences, Wuhan, China

**Keywords:** SARS-CoV-2, Nsp5, MAVS, SUMOylation, NF-κB, cytokine

## Abstract

The COVID-19 is an infectious disease caused by SARS-CoV-2 infection. A large number of clinical studies found high-level expression of pro-inflammatory cytokines in patients infected with SARS-CoV-2, which fuels the rapid development of the disease. However, the specific molecular mechanism is still unclear. In this study, we found that SARS-CoV-2 Nsp5 can induce the expression of cytokines IL-1β, IL-6, TNF-α, and IL-2 in Calu-3 and THP1 cells. Further research found that Nsp5 enhances cytokine expression through activating the NF-κB signaling pathway. Subsequently, we investigated the upstream effectors of the NF-κB signal pathway on Nsp5 overexpression and discovered that Nsp5 increases the protein level of MAVS. Moreover, Nsp5 can promote the SUMOylation of MAVS to increase its stability and lead to increasing levels of MAVS protein, finally triggering activation of NF-κB signaling. The knockdown of MAVS and the inhibitor of SUMOylation treatment can attenuate Nsp5-mediated NF-κB activation and cytokine induction. We identified a novel role of SARS-CoV-2 Nsp5 to enhance cytokine production by activating the NF-κB signaling pathway.

## Introduction

The emerging infectious disease COVID-19 caused by the severe acute respiratory symptom coronavirus-2 (SARS-CoV-2) has caused a global pandemic. COVID-19 has been characterized by lymphopenia and elevation pro-inflammatory cytokines, especially in severe cases and in the elderly population, leading to acute respiratory distress syndrome (ARDS), multiple organ failure, and other symptoms that endanger health and survival (1–3). Although tens of thousands of scientists are working intensively on the pathogenesis of SARS-CoV-2, there is still no effective treatment for COVID-19 to date. Therefore, it is necessary to accelerate insight into the pathogenic molecular pathways elicited as a consequence of SARS-CoV-2 infection and elucidate the interaction between the virus and host.

SARS-CoV-2 is a positive stranded RNA virus with an envelope and belongs to the family *Coronaviridae*. One-third of its genome near the 3’ terminus encodes four structural proteins, and about two-thirds of the genome near the 5’ terminus encode 16 non-structural proteins (Nsp1-16) (4, 5). Several Nsps are known to act on various enzymes required for the viral replication and transcription process. In infected cells, viral proteins play important roles in the interaction between the virus and host (6). In our screening system, we found that Nsp5 can enhance cytokine expression. Nsp5, also known as 3-Chymotrypsin-like protease (3CL^pro^), is a cysteine protease that hydrolyses viral polyproteins to produce functional proteins Nsp4-Nsp16 in the virus replication, which plays an indispensable role in the virus life cycle (7). SARS-CoV Nsp5 was reported to induce the secretion of chemokines including CCL5, CXCL10, and CCL3 in human lung epithelial cells (8).

The expression of chemokines and cytokines is regulated by numerous transcription factors. The nuclear factor kappa-light-chain enhancer of activated B cells (NF-κB) is a transcription factor that regulates multiple aspects of the immune function and mediates the inflammatory response. In general, the NF-κB signaling involves the phosphorylation and degradation of the IκB kinase beta (IKKβ), thereby leading to the liberation of the p50 and p65 dimers. Subsequently, these subunits translocate into the nucleus, where they act as potential transcriptional factors to activate the transcription of different genes involved with inflammation, cell proliferation, and apoptosis. Therefore, NF-κB is the primary transcription factor that regulates numerous cellular responses including early innate immunity, chronic inflammatory states, viral infections, septic shock syndrome, and multi-organ failure (9–11). Hence, it is interesting to investigate whether SARS-CoV-2 Nsp5 could induce pro-inflammatory cytokines through the NF-κB pathway.

Innate immunity is an evolutionarily conserved defense mechanism against microbial infections. In higher organisms, viral infections trigger various signaling pathways by utilizing distinct adaptor proteins, kinases, and regulatory proteins (12). The mitochondrial antiviral-signaling protein (MAVS) activation is a key event of signaling pathways following infection with RNA viruses when viral RNA is recognized by the RIG-I pattern recognition receptors (PRR), ultimately leading to the induction of antiviral and inflammatory responses mediated by type I interferon (IFN) and NF-κB pathways (13). Several reports suggest that MAVS is an important target of the viral protein to execute the pathogenicity of virus. The expression and function of MAVS are tightly regulated *via* ubiquitination and phosphorylation at post-translational levels (14, 15). The hepatitis B virus (HBV) protein X (HBX) binds to MAVS and promotes its ubiquitination at Lys136, leading to proteasomal degradation (16). SARS-CoV ORF-9 catalyzes the K48-linked ubiquitination and degradation of the MAVS to limit interferon production (17). The VP3 protein of rotaviruses mediates the phosphorylation of MAVS, leading to its proteasomal degradation (18). Recently, a study reported that SARS-CoV-2 ORF9b colocalizes and interacts with MAVS and inhibits MAVS-induced IFN activation (19).

SUMOylation has been identified as an evolutionarily conserved ubiquitin-like post-translational modification in key cellular processes that regulates numerous biological processes, such as protein subcellular localization or stability, transcription, DNA repair, innate immunity, or antiviral defense (20). Four different SUMO isoforms have been described in mammals (SUMO1 to SUMO3). The SUMO1 protein is found mainly conjugated to targets due to its limited quantity and only shares 50% identity with SUMO2/3. SUMO2 and SUMO3 share 97% sequence identity and often are referred to as SUMO2/3 (21, 22). SUMO has a critical role in the signaling pathway of innate immunity. By conjugating MDA5 and RIG-I, SUMO inhibits their phosphorylation and induces their stabilization, leading to the activation of IFN production (23). The conjugation of SUMO to NEMO inhibits its interaction with the deubiquitinase CYLD strengthening the activation of IKK (24). For the SARS-CoV-2 infection, numerous studies have profiled the effects of viral infection on ubiquitination and phosphorylation by proteomics analysis, which revealed that viral infection broadly regulated host cellular immunity factors at post-translational levels (25, 26).

In this study, we found that SARS-CoV-2 Nsp5 can elevate the expression of inflammatory cytokines by activating the NF-κB signaling pathway. Further studies illustrate that Nsp5 can increase the stability of MAVS by promoting its SUMOylation, leading to increased levels of MAVS protein and activation of NF-κB signaling pathway. This study provides new and diverse mechanistic insights into the relationship between SARS-CoV-2 infection and host immune response, which vastly expands the functional diversity of Nsp5 in the modulation of MAVS response and inflammatory cytokine expression.

## Materials and Methods

### Cell Culture and Transfection

HEK293T cells, Vero E6 cells and Calu-3 cells were grown in Dulbecco’s modified Eagle medium (DMEM, Gibco, Logan, UT, USA) supplemented with 10% fetal bovine serum (Gibco, Logan, UT, USA) at 37°C in a 5% CO_2_ incubator. THP-1 cells were cultured in RPMI-1640 medium (Gibco, Logan, UT, USA) containing 10% FBS. To induce monocyte/macrophage differentiation, THP-1 cells at a density of 2.5 × 10^5^ cells/cm^2^ were added with 100 nM Phorbol-12-myristate-13-acetate (PMA, Cat#P8139, Sigma Aldrich, St. Louis, MO, USA) and cultured for 48 h at 37°C. Plasmid transfection to cells was performed using lipofectamine 2000 reagent (Invitrogen, Carlsbad, CA, USA) according to the manufacturer’s instructions.

### hPBMC Isolation

Whole blood was collected from healthy donors in tubes containing EDTA, according to the manufacturer’s instructions. Approval was obtained from the School of Medicine, Jianghan University (Approval number: YXLL2021-004). Written informed consent was obtained from each participant. Human peripheral blood mononuclear cells (hPBMCs) were isolated from peripheral blood by apheresis and density gradient separation. 2× 10^6^ cells/mL were then seeded in 6-well plates that were pre-coated with poly-L-ornithine (Sigma, St. Louis, MO). Cells were incubated for 7 days in media containing macrophage colony-stimulatory factor (M-CSF) (Sigma, St. Louis, MO). Cells were then transfected with Flag-Nsp5 or Vector for 24h. Thereafter, medium was collected and centrifuged to obtain culture supernatants for ELISA detection, and the cells were lysed for PCR detection.

### Plasmids, Reagents, and Antibodies

Fragments of Nsp5 were obtained from the cDNA of SARS-CoV-2 (IVCAS 6.7512). Then, Nsp5 was cloned into pCMV-Flag with EcoR I and Xho I sites. pNF-κB-Luc and pAP1-Luc were purchased from Miaoling Plasmid Sharing Platform (Cat#P4103/P1044, Wuhan, China); pCMV-HA-Sumo1 and pCMV-HA-Sumo2/3 were generously provided by Hanzhong Wang (Wuhan Institute of Virology, Chinese Academy of Sciences, Wuhan, China) (27). For MAVS RNAi, the corresponding double-stranded oligonucleotides of MAVS shRNA sequence and control shRNA were generated by GeneChem (Shanghai, China) and inserted into pU6-EGFP-puromycin.

MLN120B (Cat#HY-15473) was purchased from MedChemExpress (Shanghai, China); cycloheximide (CHX, Cat#ab120093) and Ginkgolic acid (Cat#ab142629) were obtained from Abcam (Cambridge, MA, USA).

The antibodies used are listed in **Table 1**.

**Table 1:** Antibodies used in this study.

Antibodies	SOURCE	IDENTIFIER
IL-1β	Cell Signaling Technology	Cat# 12703S
SUMO-1	Cel l Signaling Technology	Cat#4930S
SUMO-2/3	Cell Signaling Technology	Cat#4971S
p50	Proteintech	Cat#14220-1-AP
MAVS	Proteintech	Cat#14341-1-AP
Flag	Proteintech	Cat#20543-1-AP
Actin	Proteintech	Cat#66009-1-Ig
p65	Beyotime	Cat# AF5243
p-p65	Beyotime	Cat# AF5881
IKBα	Beyotime	Cat# AI096
p-IKBα	Beyotime	Cat# AF1870
IKKβ	Beyotime	Cat# AF7200
p-IKKα/β	Beyotime	Cat# AF5839
TRAF2	Beyotime	Cat# AF5327
TRAF6	Beyotime	Cat# AF8223
RIG-I	Beyotime	Cat# AF7890
Tomm20	Beyotime	Cat# AF1717
HA	Absin	Cat# abs137963
Lamin B	Absin	Cat# abs149754
SARS-CoV-2-NP	Sino Biological	Cat#40143-MM05

### Virus Infection

The SARS-CoV-2 (IVCAS 6.7512) was propagated in Vero E6 cells and viral titer (TCID_50_) was determined on Vero E6 cells (28). Calu-3 cells were incubated with SARS-CoV-2 for 1 hour at a multiplicity of infection (MOI) of 1. After removing the infectious liquid, cells were washed with PBS and maintained in culture medium. At 24 h post-infection, cells were lysed and subjected to western blot detection. All viral infection operation were conducted under BSL3 in Wuhan Institute of Virology, Chinese Academy of Sciences.

### Dual-Luciferase Reporter Activity Assays

NF-κB or AP-1 firefly luciferase reporter plasmid (0.25 μg), control *Renila* luciferase plasmid (0.01 μg) and Flag-Nsp5 or Vector (0.25 μg) were co-transfected to cells using lipofectamine 2000. This mixture of plasmids was then added to each well of 24-well plates with HEK293T cells growing at 70% confluence. After 36 h, the cells were harvested for luciferase activity assays using the dual-luciferase reporter assay kit (Promega, Madison, WI, USA).

### Immunofluorescence Staining

Flag-Nsp5 transfected Calu-3 cells were examined for NF-κB localization by the immunofluorescence method. Cells were cultured on sterile cover slides overnight and fixed by 4% methanal. Samples were blocked with 5% BSA and 0.1% Triton X-100, then incubated with rabbit polyclonal antibody against p50 at 1: 100 dilution overnight at 4°C After rinsing with TBST, sections were incubated with secondary goat IgG Alexa Fluor 488-conjugated anti-rabbit antibody (Invitrogen, Carlsbad, CA, USA) for 1 h at room temperature. Nuclei were stained with Hoest33342 (Sigma Aldrich, St. Louis, MO, USA). Images were captured using a fluorescence microscope (BX51, Olympus).

### RNA Isolation and PCR Analysis

Total RNA was extracted with the Trizol reagent (Invitrogen, Carlsbad, CA, USA), and 2 μg RNA was reverse transcribed using the MMLV Reverse Transcriptase (Takara Bio, Tokyo, Japan). The obtained cDNAs were used as templates for quantitative real-time PCR (qRT-PCR) or PCR amplification to determine the mRNA expression levels specific primers. The housekeeping GAPDH gene was used for normalization. Quantitative real-time PCR was performed using the following primer sets:

IL-1β forward: 5’-CCAGGGACAGGTATGGAGCA-3’, reverse: 5’-TTCAACACGCAGGACAGGTACAG-3’; IL-2: 5’-CAACTCCTGTCTTGCATTGCACTAA-3’, reverse: 5’-AATGTGAGCATCCTGGTGAGTTTG-3’; IL-6 forward: 5’-AAGCCAGAGCTGTGCAGATGAGTA-3’, reverse: 5’-TGTCCTGCAGCCACTGGTTC-3’; TNF-α forward: 5’-CCTCTCTCTAATCAGCCCTCTG-3’, reverse: 5’- GAGGACCTGGGAGTAGATGAG-3’; MAVS forward: 5’- CGGGAGCAGCAGAAATGA-3’, reverse: 5’- GGCAAGATCCTCGAAGCAG-3’; Nsp5 forward: 5’- TGACAGGCAAACAGCACAAG-3’, reverse: 5’- AGCAGCGTACAACCAAGCTAA-3’;and GAPDH forward: 5’-GCACCGTCAAGGCTGAGAAC-3’, reverse: 5’-ATGGTGGTGAAGACGCCAGT-3’, using TB Green^®^ Premix Ex Taq™ II (Takara Bio, Tokyo, Japan). PCR reactions were performed using the qRT-PCR CFX 96 Thermocycler (Bio-Rad Laboratories, Inc., CA, USA) in a total volume of 20 µL. The relative amount of PCR products was calculated using the 2^− ΔΔCt^ method.

### Chemical Treatment

MLN120B was prepared as 10 mM stock in dimethyl sulfoxide, stored at -20°C, and diluted at a final concentration of 10 or 5 µM in culture medium immediately before use. Cycloheximide was prepared as 20 mg/ml stock in dimethyl sulfoxide, and diluted at a final concentration of 50 µg/ml. Ginkgolic acid was prepared as 10 mM stock in dimethyl sulfoxide, and diluted at a final concentration of 5 µM.

### Co-Immunoprecipitation

Transfected cells were harvested and lysed in 200 µl IP lysis buffer containing protease inhibitors. For SUMOylation assays, 20 mM N-ethylmaleimide (NEM) (#E3876, Sigma Aldrich, St. Louis, MO, USA) was added to inhibit de-SUMOylation enzymes. Then, lysates were clarified by centrifugation (13,000 *g* for 15 min at 4°C). Clarified lysates (150 µl) were incubated with HA-Agarose beads (#A2095, Sigma Aldrich, St. Louis, MO, USA) with rotation overnight at 4°C. The lysates and beads were centrifuged at 8,000 *g* for 1 min, the supernatant was removed and the pelleted beads were washed three times with TBS buffer (50 mM Tris, 150 mM NaCl, pH 7.5). The immunoprecipitants were eluted by boiling with 2× SDS sample buffer and analyzed by western blot analysis. For the rests of the lysates was analyzed by western blot as input whole cell lysis (WCL).

### Western Blot

The protein contents of the samples were supplemented with protease and phosphatase inhibitors, and determined using the BCA kit (Beyotime Biotechnology, Beijing, China). Then, 30 µg of the cell lysate was separated by 10% sodium dodecyl sulfate polyacrylamide gel electrophoresis (SDS-PAGE), and the separated proteins were transferred onto a polyvinylidene fluoride (PVDF) membrane (Millipore, MA, USA). The membranes were blocked with 5% nonfat milk for 1 h and then incubated with primary antibodies overnight at 4°C. Subsequently, the membranes were incubated with appropriate HRP-conjugated secondary antibodies for 1 h at room temperature. Bands were visualized with ECL Substrate (Millipore, MA, USA) using the Chemidoc XRS Gel Imaging System (Bio-Rad Laboratories, Inc., CA, USA). Each band was quantified by densitometry using Image J.

### ELISA

Levels of IL-6 in the supernatants were determined using human ELISA™ kit (Beyotime Biotechnology, Beijing, China) according to the manufacturer’s instructions. Concentrations of IL-6 in supernatants were calculated from a human IL-6 standard curve, and the assay range was 18.75-600 pg/mL. Similarly, levels of IL-1β were evaluated using human IL-1β ELISA kit (Bio-Swamp, Wuhan, China) with a range of detection of 12.5-1000 pg/mL.

### Extraction of Cellular Components

The extraction of cytoplasmic and nuclear proteins was prepared using the Nuclear and Cytoplasmic Protein Extraction Kit (Beyotime Biotechnology, Beijing, China) according to the manufacturer’s protocol. Mitochondrial and cytosolic fractions prepared using the Cell Mitochondria Isolation Kit (Beyotime Biotechnology, Beijing, China). Briefly, cells were incubated in cell lysis buffer at 4°C and disrupted with a glass tissue grinder. The cell lysate was centrifuged at 800 g for 10 min, and the supernatant was further centrifuged at 10,000 *g* for 20 min. The resulting final supernatants and pellets contained the cytosolic and mitochondrial fractions, respectively.

### Statistical Analysis

All data were presented as average values and standard errors (Mean ± SEM). All assays were conducted in triplicate. The analysis of variance (ANOVA) followed by Dunnett’s test and Student’s two-tailed t-test were used to determine statistically significant differences using GraphPad Prism 5 software (GraphPad Software, Inc., USA); the criterion for significance was set at *P* < 0.05.

## Results

### SARS-CoV-2 Nsp5 Enhances Expression of Pro-Inflammatory Cytokines in Calu-3 and THP1/PMA Cells

**Figure 1:**
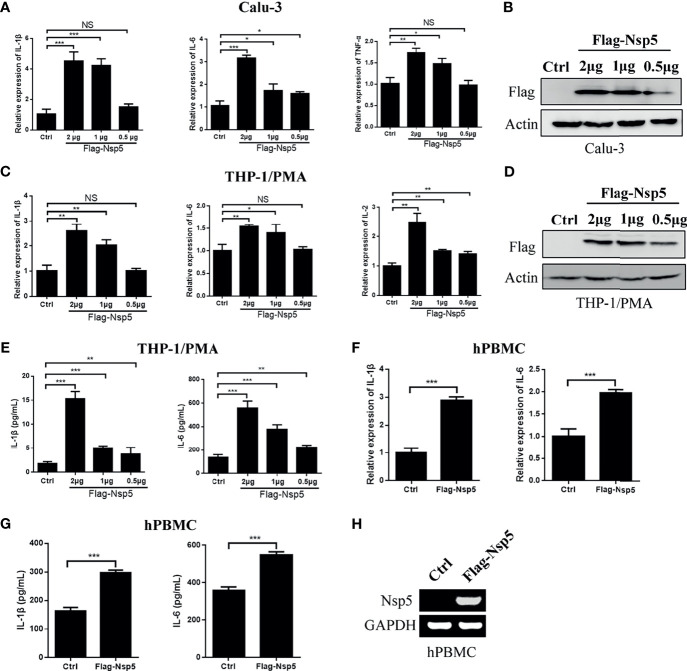
SARS-CoV-2 Nsp5 induced pro-inflammatory factors in Calu-3 and THP-1/PMA cells. Plasmid of empty vector or Flag-Nsp5 was transfected into Calu-3 **(A, B)** and THP-1/PMA cells **(C–E)**. At 24 h post transfection, the cells were harvested and the mRNA levels of IL-1β, IL-6, TNF-α, and IL-2 were determined by qRT-PCR analysis. The Flag-Nsp5 expression levels were detected by western blot. The secretion levels of IL-1β and IL-6 in the culture supernatant were determined with ELISA analysis. **(F, G)** Vector or Flag-Nsp5 was transfected into the hPBMC. At 48 h post transfection, the cells were harvested and the mRNA levels of IL-1β and IL-6 were determined by qRT-PCR analysis. The secretion levels of IL-1β and IL-6 in the culture supernatant were determined with ELISA analysis. **(H)** The expression of Flag-Nsp5 was determined by PCR. The data were presented as Mean ± SEM for three biological replicates, and statistical significance was calculated by one-way ANOVA, *P < 0.05, **P < 0.01, ***P < 0.001, NS, not significant.

SARS-CoV-2 predominantly infects airway and lung tissue in infected individuals and leads to an excessive inflammatory response associated with disease severity. Therefore, we transfected Nsp5 expression plasmids in Calu-3 cells, which are lung cells that can be infected by SARS-CoV-2, and found the mRNA levels of IL-1β, IL-6, and TFN-α were increased in a Nsp5 dose-dependent manner (**Figures 1A, B**). Because macrophages are the important cytokine-producing cells and also the target cells of SARS-CoV-2, we also detected the cytokine expression in the Nsp5 overexpressed THP-1/PMA cells. Consistent with the results in Calu-3 cells, the mRNA levels of IL-1β, IL-6, and IL-2 increased with Nsp5 dose-dependent activation (**Figures 1C, D**). The results show that the mRNA expression level of IL-1β was increased 4.2-fold in Calu-3 cells and 2.5-fold in THP1/PMA cells after 2 μg Nsp5 plasmid transfection. Meanwhile, the secretion levels of IL-6 and IL-1β in the culture supernatant were determined with ELISA analysis and the results showed that the expression levels of IL-6 and IL-1β were also increased with Nsp5 dose-dependent activation (**Figure 1E**). Moreover, hPBMCs were prepared from healthy donors and transfected with Flag-Nsp5 (**Figure 1H**). Overexpression of Flag-Nsp5 led to upregulation of mRNA expression levels of IL-1β and IL-6 with qRT-PCR analysis and increased the levels of IL-1β and IL-6 in the culture supernatant determined with ELISA analysis in the hPBMC (**Figures 1F, G**). The above results illustrate that SARS-CoV-2 Nsp5 can induce pro-inflammatory cytokine expression, especially IL-1β, in both lung cells and macrophages.

### Nsp5 Induces NF-κB Activation

**Figure 2:**
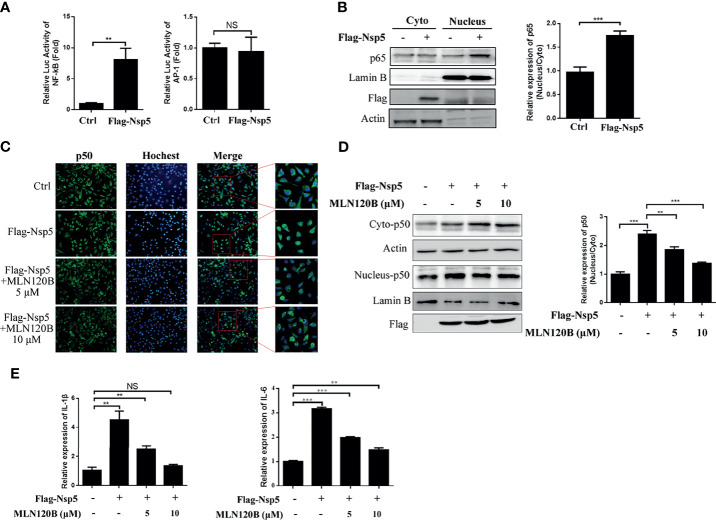
SARS-CoV-2 Nsp5 enhances cytokine expression through activating NF-κB signaling pathway. **(A)** 293T cells were co-transfected with the reporter vectors of NF-κB or AP-1 with Flag-Nsp5 or empty vector. Cells were harvested after transfection for 36 h and assayed for dual-luciferase activity. **(B)** Calu-3 cells were transfected with Flag-Nsp5 or empty vector for 36 h, the cytosol and nucleus fractions of cells were prepared. The protein p65, Flag-Nsp5, Lamin B (loading control of nucleus) and Actin (loading control of cytoplasm) were analyzed by western blot. The relative expression levels of p65 in nucleus to p65 in cytoplasm were estimated by densitometry calculation. **(C–E)** Calu-3 cells were transfected with an empty or Flag-Nsp5 vector for 36 h and treated with inhibitor MLN120B for 12 h before cells were harvested with a final concentration of 5 or 10 μM. Then, cells were immuno-stained with anti-p50 antibody (green) and counterstained with Hoest33342 to examine chromosomes (blue) **(C)**. Expression of p50 protein in cytosolic and nuclear were detected by western blot and the relative expression levels of p50 in nucleus to p50 in cytoplasm were estimated by densitometry calculation **(D)**. mRNA levels of IL-1β and IL-6 measured with qRT-PCR **(E)**. Data are presented as Mean ± SEM for three biological replicates, and statistical significance was calculated by one-way ANOVA, **P < 0.01, ***P < 0.001, NS, not significant.

The cytokine expression was regulated by numerous transcription factors, including NF-κB and AP-1. Therefore, to investigate which transcription factor was activated after Nsp5 overexpression, Flag-Nsp5 was transfected into HEK293T along with a NF-κB or AP-1 reporter plasmid. The results show that the luciferase activities of NF-κB reporter plasmid were increased more than seven-fold in Flag-Nsp5 expression cells compared with the control, but the activity of AP-1 reporter plasmid did not change significantly in comparison (**Figure 2A**), indicating that Nsp5 might induce cytokine expression by activating NF-κB, but not the AP-1 signaling pathway. NF-κB translocating into the nucleus is the prerequisite for its activation. We detected the protein expression of p65, a subunit of NF-κB, both in the cytoplasm and nucleus of Nsp5 overexpressed Calu-3 cells. Western blot analysis shows that the nucleus-p65 had a higher level of Nsp5 expression than the control (**Figure 2B**). To further confirm that Nsp5 can induce NF-κB activation, we added a NF-κB pathway inhibitor MLN120B, which is an effective IKKβ inhibitor and block NF-κB pathway, in Flag-Nsp5 plasmid transfected Calu-3 cells. Immunofluorescence and western blot analysis showed that p50, another subunit of NF-κB, had a higher expression level in nucleus of Nsp5 overexpressed cells than that in the control, and the promotion of p50 in nuclear (nucleus-p50) caused by Nsp5 was almost eliminated in the MLN120B treatment cells (**Figures 2C, D**). Finally, to confirm whether the Nsp5-enhanced cytokines are dependent on NF-κB activation, the mRNA levels of IL-1β and IL-6 were measured with or without MLN120B treatment. As shown in **Figure 2E**, MLN120B treatment strongly inhibits the induction of IL-1β and IL-6 mRNA expression, suggesting that activation of NF-κB was essential for the up-regulation of cytokines by Nsp5.

### Nsp5 Activates NF-κB by Enhancing Protein Levels of MAVS

**Figure 3:**
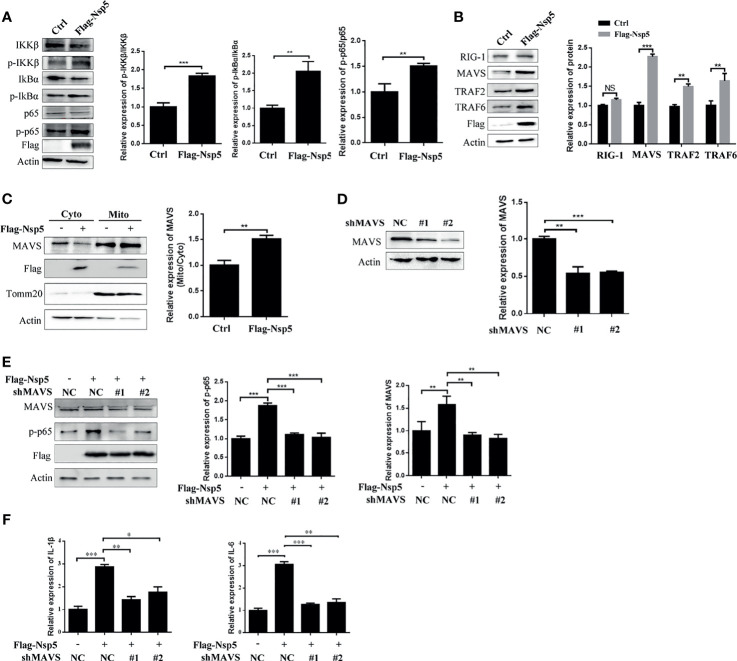
SARS-CoV-2 Nsp5 promotes IL-1β expression through targeting MAVS. **(A, B)** Calu-3 cells were transfected with Flag-Nsp5 or empty vector for 36 h, and protein levels were analyzed by western blot. The relative expression levels of p-IKKβ/IKKβ, p-IkBα/IkBα and p-p65/p65 **(A)** and relative expression levels of proteins to Actin **(B)** were estimated by densitometry calculation. **(C)** Calu-3 cells transfected with Flag-Nsp5 or empty vector for 36 h. Then, cytosol and mitochondrial fractions of transfected cells were prepared, the levels of MAVS protein, Flag-Nsp5, Tomm20 (loading control of mitochondria) and Actin (loading control of cytoplasm) were analyzed by western blot. The relative expression levels of MAVS in mitochondria to MAVS in cytoplasm were estimated by densitometry calculation. **(D)** Calu-3 cells were transfected with MAVS-shRNAs or negative control for 36 h. The levels of mRNA and protein of MAVS were analyzed by qRT-PCR or western blot, respectively. The relative expression levels of MAVS to Actin were estimated by densitometry calculation. **(E, F)** Calu-3 cells were co-transfected with MAVS-shRNAs or negative control with Flag-Nsp5 or empty vector for 36 h, the levels of MAVS, p-p65, Flag-Nsp5, and Actin (loading control) were analyzed by western blot and relative expression levels of MAVS and p-p65 to Actin were estimated by densitometry calculation **(E)**. mRNA levels of IL-Iβ and IL-6 were analyzed by qRT-PCR **(F)**. Data were presented as Mean ± SEM for three biological replicates, and statistical significance was calculated by one-way ANOVA, *P < 0.05, **P < 0.01, ***P < 0.001, NS, not significant.

To further elucidate the molecular basis of NF-κB activation by Nsp5 protein, we investigated the upstream effectors of the NF-κB signal pathway. First, the phosphorylation of IKKβ and IkBα, which is followed NF-κB activation and transportation to nucleus, was detected by a western blot in Nsp5 overexpressed Calu-3 cells. As shown in **Figure 3A**, Nsp5 increased the levels of p-IKKβ and p-IkBα. Furthermore, based on findings from other coronaviruses, the most likely PRR involved in recognizing SARS-CoV-2 are TLR in the endosome or the cytosolic sensors RIG-1 and MDA5 (29, 30). Meanwhile, a recent study found that SARS-CoV-2 is sensed by RIG-1 rather than MDA5, and initiates the MAVS-dependent IFN signaling pathway (31). MAVS, mainly located in mitochondria, can play a crosstalk role between the IFN and NF-κB pathway through tumor necrosis factor receptor-associated factors (TRAFs) (14). Therefore, we detected the above factors, RIG-1, MAVS, TRAF2, and TRAF6, upstream of NF-κB signal pathway in Nsp5 overexpressed Calu-3 cells. Surprisingly, Nsp5 increased the protein level of MAVS rather than RIG-1 (**Figure 3B**), and downstream proteins TRAF2 and TRAF6 were also increased. The expression level of MAVS in mitochondria likewise increased after Nsp5 overexpression (**Figure 3C**). To further investigate whether MAVS was required for cytokine induction, two shRNAs targeting different MAVS mRNA regions were transfected. As shown in **Figure 3D** using the western blot and qRT-PCR methods, two shMAVS could significantly reduce the expression of endogenous MAVS. In MAVS knockdown cells, Nsp5 does not enhance the protein level of p-p65 and mRNA level of IL-1β and IL-6 (**Figures 3E, F**). In summary, these results suggest that Nsp5-induces the expression of cytokines by targeting the MAVS protein.

### Nsp5 Induced Cytokines by Promoting the SUMOylation of MAVS

**Figure 4:**
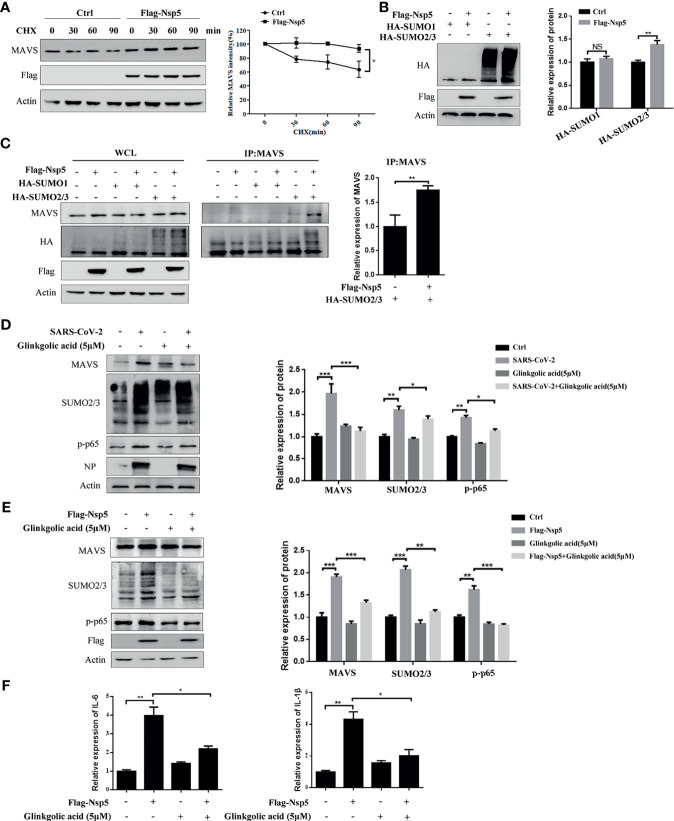
Nsp5 drives expression of IL-1β by enhancing SUMOylation of MAVS. **(A)** Calu-3 cells were transfected with Flag-Nsp5 or empty vector for 36 h and then treated with CHX for different times before harvesting. Then, the levels of MAVS, Flag-Nsp5, and Actin were analyzed by western blot. The relative expression levels of MAVS to Actin were estimated by densitometry calculation. **(B)** Calu-3 cells were co-transfected with the indicated plasmid for 36 h and the levels of HA-SUMOs, Flag-Nsp5, and Actin (loading control) were analyzed by western blot. The relative expression levels of proteins to Actin were estimated by densitometry calculation. **(C)** Calu-3 cells were co-transfected with the indicated plasmids for 36 h to measure SUMOylation of MAVS by Co-IP. The relative expression levels of MAVS by Co-IP to Actin were estimated by densitometry calculation. **(D)** Vero E6 cells were incubated with SARS-CoV-2 for 1 h at MOI of 1. At 24 h post-infection, the levels of MAVS, SUMO2/3, p-p65, SARS-CoV-2-NP, and Actin (loading control) were analyzed by western blot. The relative expression levels of proteins to Actin were estimated by densitometry calculation. **(E, F)** Calu-3 cells were transfected with Flag-Nsp5 or vector for 36h and treated by Glinkgolic acid and the levels of MAVS, SUMO2/3, p-p65, Flag-Nsp5, and Actin were analyzed by western blot and relative expression levels of proteins to Actin were estimated by densitometry calculation **(E)**. mRNA levels of IL-Iβ and IL-6 were analyzed by qRT-PCR **(F)**. Data were presented as Mean ± SEM for three biological replicates, and statistical significance was calculated by one-way ANOVA, *P < 0.05, **P < 0.01, ***P < 0.001, NS, not significant.

To determine whether Nsp5 affects the production or degradation of MAVS protein, Calu-3 cells transfected with Flag-Nsp5 were treated with the translation inhibitor cycloheximide (CHX). We found that the degradation of MAVS protein was delayed after Calu-3 cells were transfected Flag-Nsp5 (**Figure 4A**). These results suggest that Nsp5 inhibits the degradation of MAVS compared to the control. SUMOylation, a post-translational modification, can improve the protein stability. We thus addressed whether Nsp5 can regulate MAVS level through SUMOylation. By co-expressing SUMO proteins and Nsp5 in HEK293T cells, we found that the amount of total SUMO2/3 rather than SUMO1 was increased by Nsp5 (**Figure 4B**). Remarkably, the levels of SUMO2/3 conjugation to MAVS were increased by Nsp5, which gains the capability of protein expression of MAVS (**Figure 4C**). Next, we wondered whether SARS-CoV-2 infection affect the SUMOylation and MAVS. We infected Vero E6 cells with SARS-CoV-2 incubated with Ginkgolic acid or not, an inhibitor of SUMOylation. The cells were lysed after 24 hours and analyzed with western blot assay. The results showed that the levels of MAVS protein, endogenous SUMO2/3 and p-p65 were increased after SARS-CoV-2 infection, while treatment with Ginkgolic acid decreased the expression of MAVS, endogenous SUMO2/3 and p-p65 induced by Nsp5 (**Figure 4D**). These results suggested that SARS-CoV-2 infection could induce the SUMOylation of cell protein and the expression of MAVS, and MAVS expression might be enhanced by SUMOylation during SARS-CoV-2 infection. To further investigate whether SUMOylation of MAVS was required for Nsp5, Calu-3 cells were transfected Flag-Nsp5 and treated with or without Ginkgolic acid. The western blot assay shows that treatment with Ginkgolic acid decreased Nsp5-induced the expression of MAVS, SUMO2/3 and p-p65 (**Figure 4E**). Meanwhile, the mRNA levels of IL-1β and IL-6 likewise dropped after Ginkgolic acid treatment upon Nsp5 overexpression (**Figure 4F**). These results suggest that the conjugation of SUMO2/3 to MAVS induced by Nsp5 enhances the expression of cytokines.

## Discussion

Numerous studies reported that SARS-CoV-2 infection results in the release of a large number of cytokines *in vivo* and *vitro*, including IL-1β, IL-6, IL-8, IL-18, and TNF-α (32, 33). We further found that the mRNA levels of IL-1β and IL-6 were increased in both Calu-3 cells and multiple inflammatory cell models after Nsp5 overexpression. Moreover, NF-κB is a family consisting of inducible transcription factors that regulate the genes involved in immune and inflammatory responses. In a study on 28 COVID-19 patients, genes that are involved in the NF-κB signaling pathway were found to be upregulated, along with increased levels of cytokines and inflammatory markers (34). In Calu-3 cells, SARS-CoV-2 infection led to the induction of inflammatory responses and the release of multiple cytokines by promoting the activation of NF-κB signaling pathway by recruiting and activating the TAK1 and IKK complex, which is regulated by the SARS-CoV-2 N protein (35). Nsp14 hijacked IMPDH2 for NF-κB activation, contributing to abnormal inflammatory responses (36). Our study revealed that SARS-CoV-2 Nsp5 could positively regulate inflammatory responses by promoting NF-κB activation by maintaining the stability of MAVS. Some studies found that certain natural products or drugs could inhibit the infiltration of inflammatory cells and the secretion of inflammatory cytokines by inhibiting protease activity of Nsp5 (37, 38), which also implied that Nsp5 could indeed induce the expression of inflammatory cytokines. However, it needs further study the relationship between Nsp5 and inflammation in clinical research. Anyhow the above studies illustrated that the activation of the NF-κB pathway might be caused by multiple viral proteins during the SARS-CoV-2 infection process. Meanwhile, viral proteins might target different factors of the NF-κB pathway, which together led to the activation of NF-κB and inflammatory responses.

The innate immune response has a critical role in both the detection and regulation of viral infection. The role of RIG-I/MDA5 and its downstream adaptor MAVS in response to SARS-CoV-2 infection has been reported (39), while the detailed mechanism was unclear. MAVS, localized in the mitochondrial outer membrane, acts as a central signaling molecule in the RLR signaling pathway by linking the upstream viral RNA recognition to the downstream signal activation. Several studies found that viral proteins of SARS-CoV-2 suppressed interferon production by targeting multiple components of RIG1/MDA5-MAVS-IFN signaling pathways (19, 40–42). However, few studies reported that viral proteins promoted inflammatory responses through RIG1/MDA5-MAVS-NF-κB. In this study, we found that Nsp5 could increase the level of MAVS on mitochondria, leading to NF-κB activation and initiate pro-inflammatory cytokine gene expression. These results illustrate that MAVS might play multiple functions during viral infection in different pathways, which was regulated by different viral proteins to participate in the innate immune response. Furthermore, research showed that Nsp5 is located in both the nucleus and cytoplasm. Interestingly, we detected the expression of Nsp5 not only in cytoplasm but also in the mitochondria. Moreover, it is well known that mitochondria provide a crucial platform for antiviral signaling (43). Monocytes from COVID-19 patients accumulate dysfunctional mitochondria and are metabolically impaired (44). Thus, we speculated that Nsp5 might be related to the function of mitochondria. However, we did not find the interaction between Nsp5 and MAVS (data not shown). Therefore, it was necessary to further study whether Nsp5 attended to impacting mitochondrial function during viral infection.

Similarly to ubiquitylation, SUMOylation is a multi-step reaction that covalently conjugates a 12-kDa SUMO protein to regulate activity, stability and subcellular localization of target proteins (22). SUMOylation of RIG-I and of MDA5 *via* PIAS2β increases antiviral type I IFN responses (45, 46). Further, SUMO-specific protease 1 (SENP1) and SENP2 regulate the activation of IRF8 and IRF3, respectively (47, 48). Infection with the influenza A virus (IAV) results in a global increase in cellular SUMOylation (49). SARS-CoV N protein can directly interact with human Ubc9 and is modified by SUMO (50, 51). Our study found that Nsp5 could enhance the SUMOylation of MAVS to increase protein stability, resulting in driving the activation of NF-κB and the expression of cytokines. Recently, a study reported that MAVS could conjugated with SUMO3 under poly(dA: dT) treatment (52). Consistently with this result, the formation of SUMO2/3-conjugated MAVS was significantly induced upon the overexpression of Nsp5. The mechanism of phosphorylation and ubiquitination of MAVS has been elucidated in detail (14). However, specific regulatory mechanisms of SUMOylation of MAVS remain unclear. SUMOylation also regulates ubiquitination enzymes and SUMO, and ubiquitin can also antagonize each other’s effects, sometimes *via* modification of the same site(s). Therefore, whether Nsp5 could affect the ubiquitination of MAVS by regulating SUMOylation still must be further explored. Meanwhile, we also found Nsp5 could increase the expression of cellular SUMO2/3, which suggests that Nsp5 may regulate host genes broadly by post-translational modifications. Moreover, a report stated that SARS-CoV-2 Nsp5, as a protease, could directly cleave the NLRP12 and exert an inhibitory effect on NF-κB of NLRP12 (53). Therefore, whether Nsp5 can regulate the NF-κB signaling pathway by degrading other substrates requires further exploration.

In summary, we report that SARS-CoV-2 Nsp5 is identified as an inducer of cytokine. Mechanically, Nsp5 promoted the conjugation of SUMO2/3 to MAVS and maintained stable of MAVS, inducing subsequent activation of the NF-κB pathway. Thus, Nsp5 positively regulates the MAVS-activated downstream signaling pathway. These findings expounded one of the roles and mechanisms of Nsp5 regulating the innate immune response.

## Data Availability Statement

The original contributions presented in the study are included in the article/**Supplementary Material**. Further inquiries can be directed to the corresponding authors.

## Ethics Statement

The studies involving human participants were reviewed and approved by the Institutional Review Board, School of Medicine, Jianghan University (Approval number: YXLL2021-004). The patients/participants provided their written informed consent to participate in this study.

## Author Contributions

BS and XS conceived and designed the experiments. WLL, JQ, QY, SZ, and QP performed the experiments. WLL, JQ, and QY prepared the original draft of the manuscript. WLL, JQ, BS, and SL participated in data analysis and writing. YL, SH, and WL provided advice and reviewed the manuscript. All authors contributed to the article and approved the submitted version.

## Funding

The work was supported by the National Natural Science Foundation of China [grant numbers 31670167, 32100131], Wuhan Science and Technology Bureau [grant numbers 2020020601012318], and Jianghan University [grant numbers 1010/08190001, 2021yb138 and 2019037].

## Conflict of Interest

The authors declare that the research was conducted in the absence of any commercial or financial relationships that could be construed as a potential conflict of interest.

## Publisher’s Note

All claims expressed in this article are solely those of the authors and do not necessarily represent those of their affiliated organizations, or those of the publisher, the editors and the reviewers. Any product that may be evaluated in this article, or claim that may be made by its manufacturer, is not guaranteed or endorsed by the publisher.
