# Supplementary material for: SARS-CoV-2 Nsp5 Activates NF-κB Pathway by Upregulating SUMOylation of MAVS
Source: Front Immunol. 2021 Nov 10;12:750969. doi: 10.3389/fimmu.2021.750969 (PMC8631293; doi:10.3389/fimmu.2021.750969)
Supplement: Supplementary file 1 [file DataSheet_1.zip › Full scans of the WB and original microscopy images.pptx]

## Slide 1
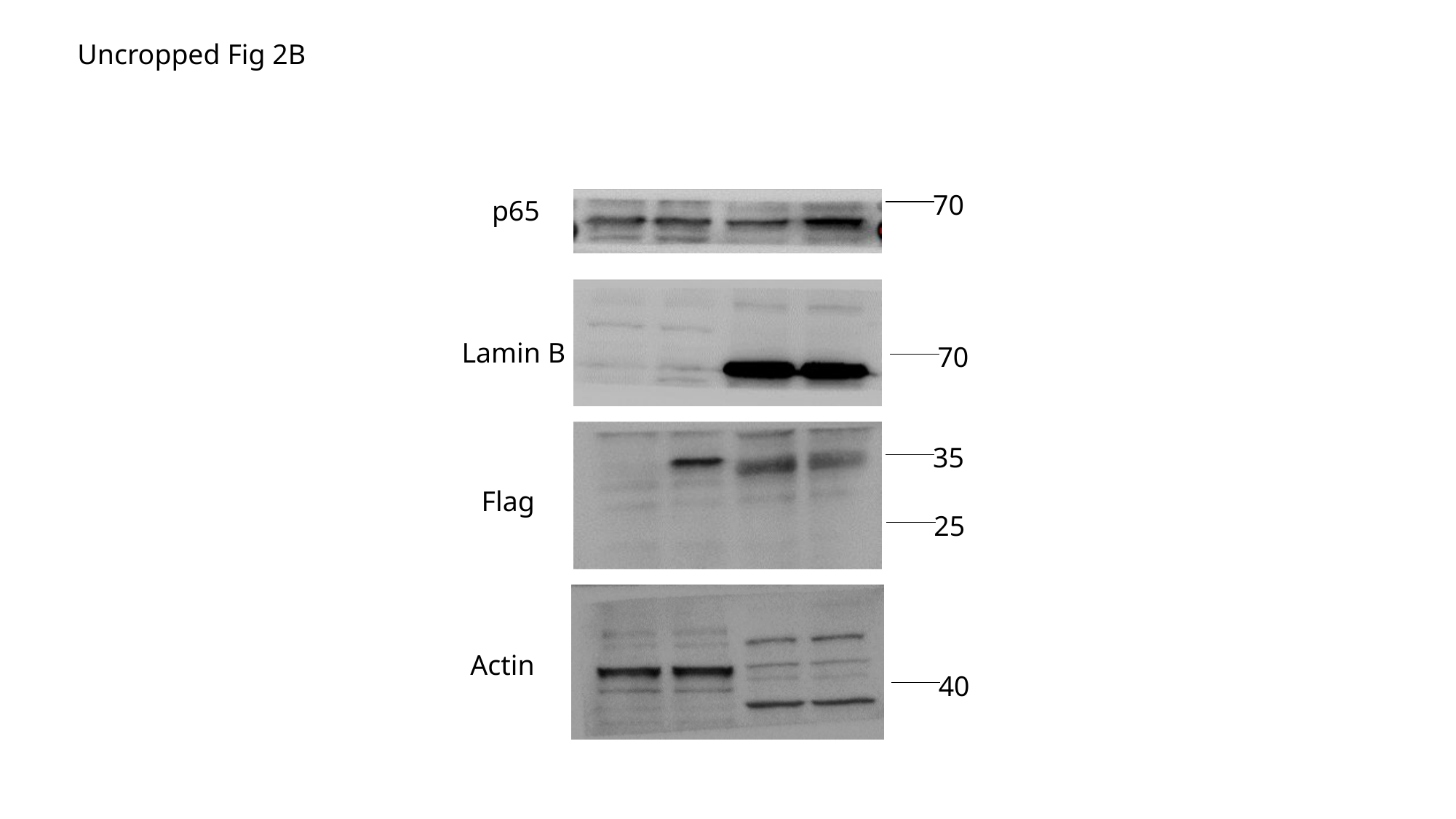

Uncropped Fig 2B
70
p65
Lamin B
70
35
Flag
25
Actin
40

## Slide 2
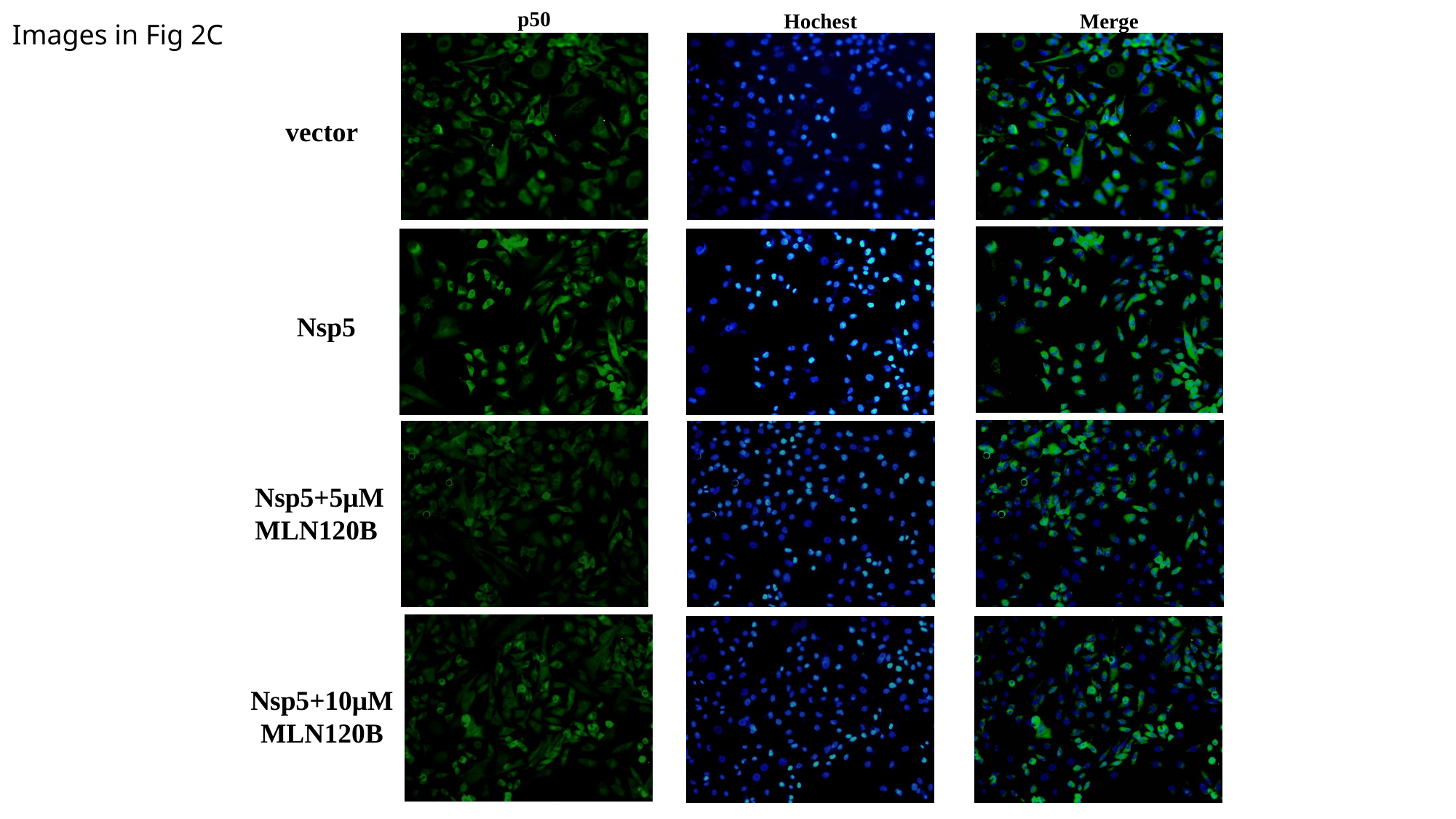

p50
Hochest
Merge
Images in Fig 2C
vector
Nsp5
Nsp5+5μM MLN120B
Nsp5+10μM MLN120B

## Slide 3
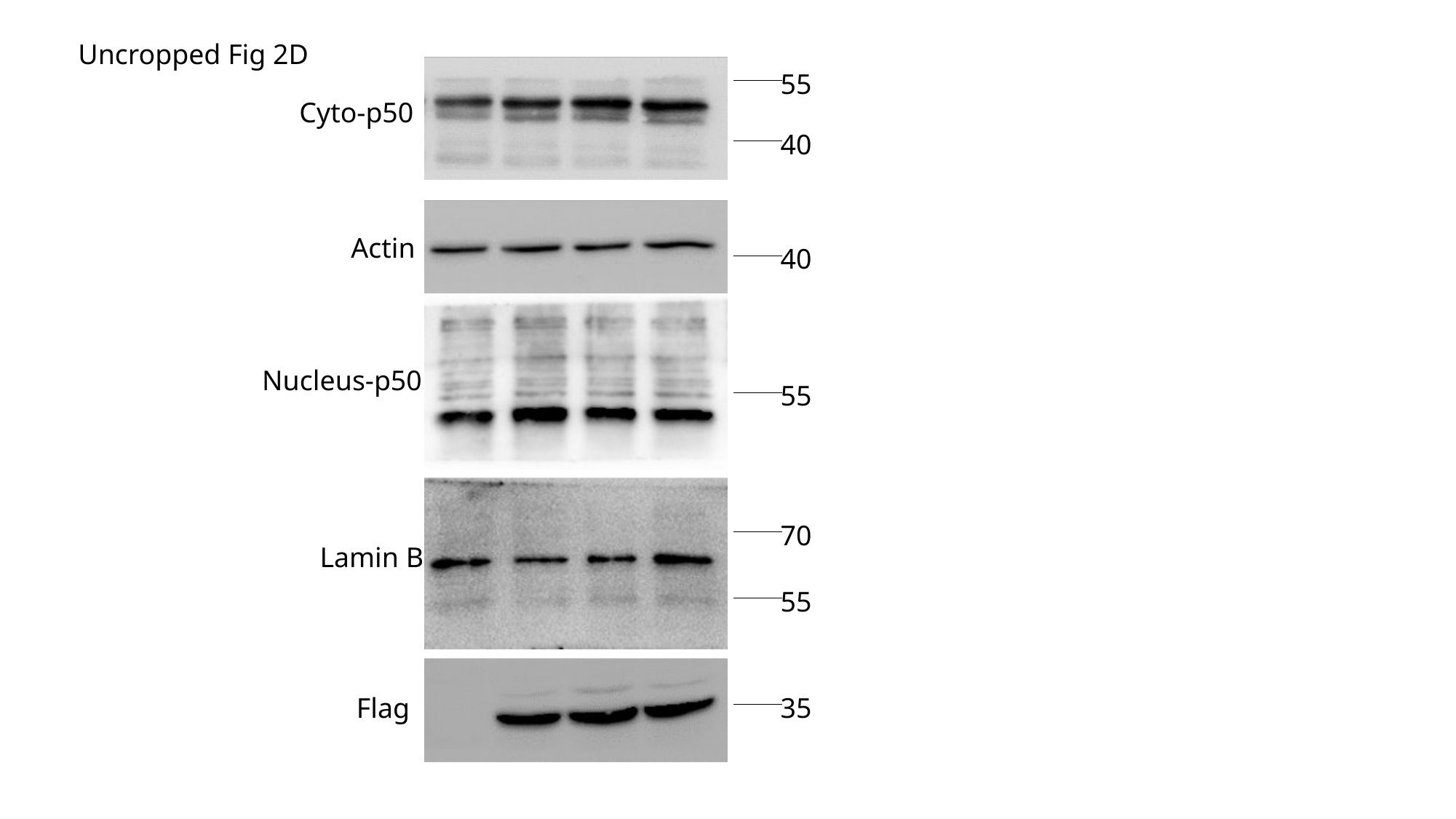

Uncropped Fig 2D
55
Cyto-p50
40
Actin
40
Nucleus-p50
55
70
Lamin B
55
Flag
35

## Slide 4
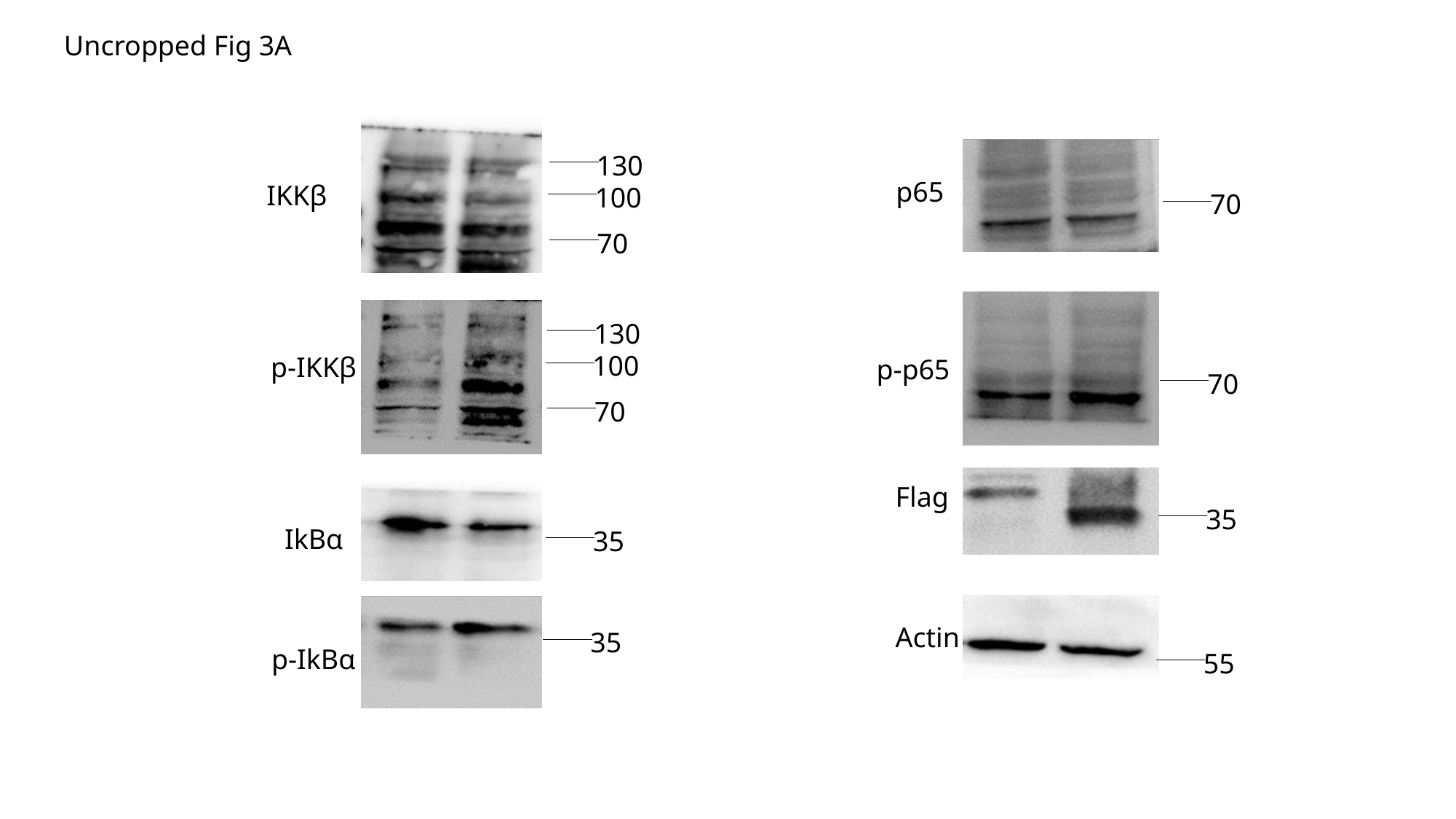

Uncropped Fig 3A
130
p65
IKKβ
100
70
70
130
100
p-IKKβ
p-p65
70
70
Flag
35
IkBα
35
Actin
35
p-IkBα
55

## Slide 5
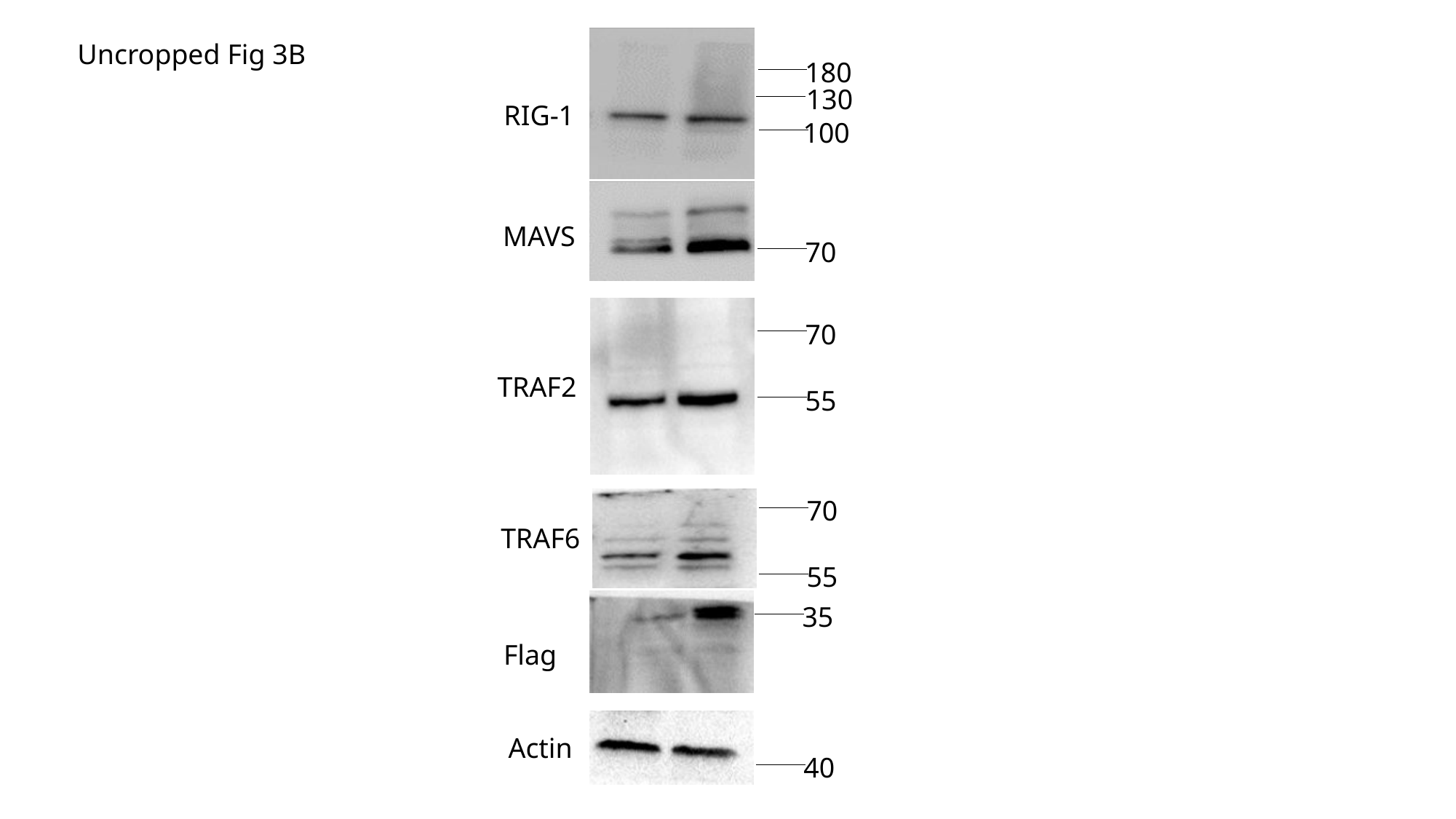

Uncropped Fig 3B
180
130
RIG-1
100
MAVS
70
70
TRAF2
55
70
TRAF6
55
35
Flag
Actin
40

## Slide 6
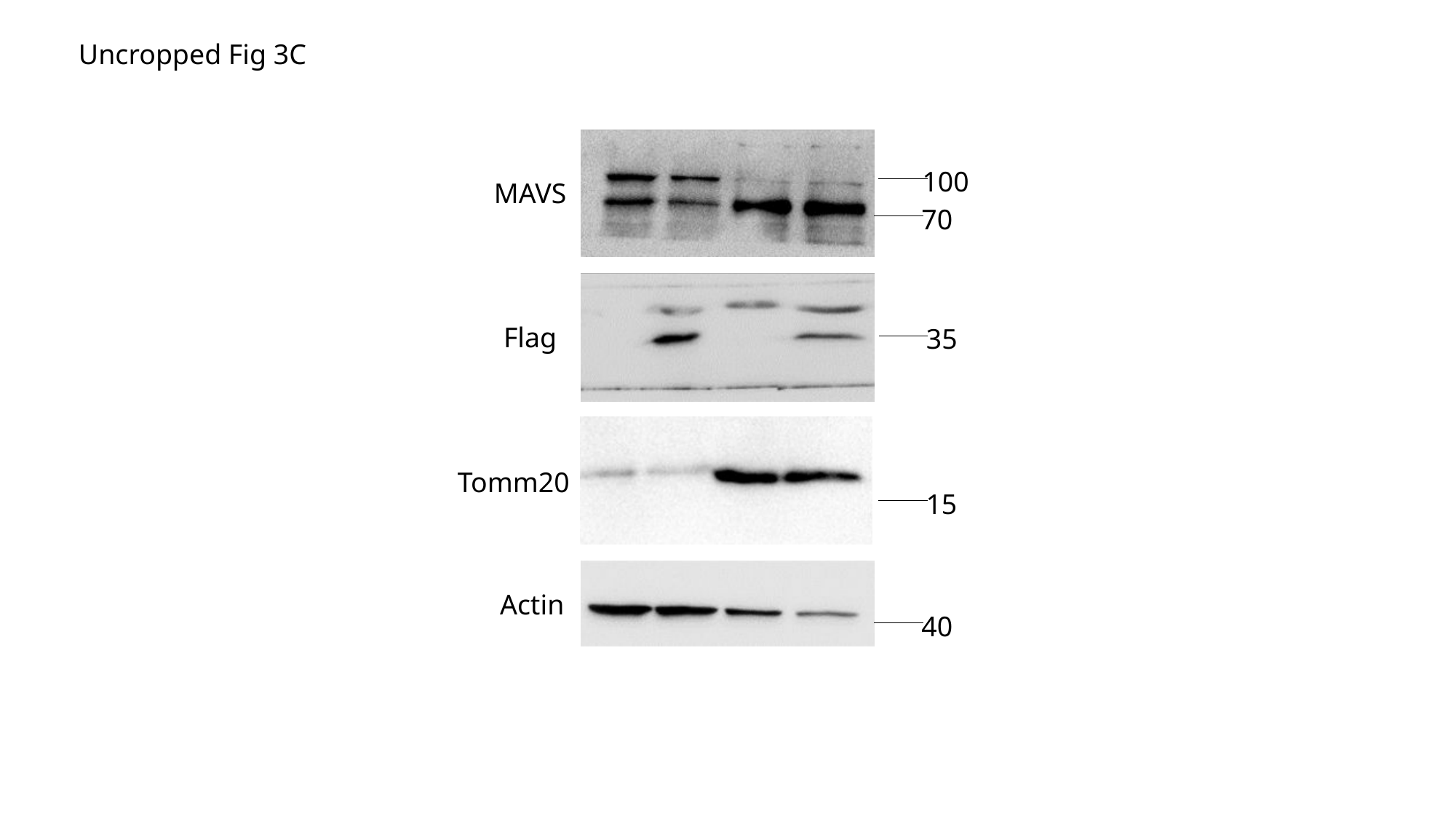

Uncropped Fig 3C
100
MAVS
70
Flag
35
Tomm20
15
Actin
40

## Slide 7
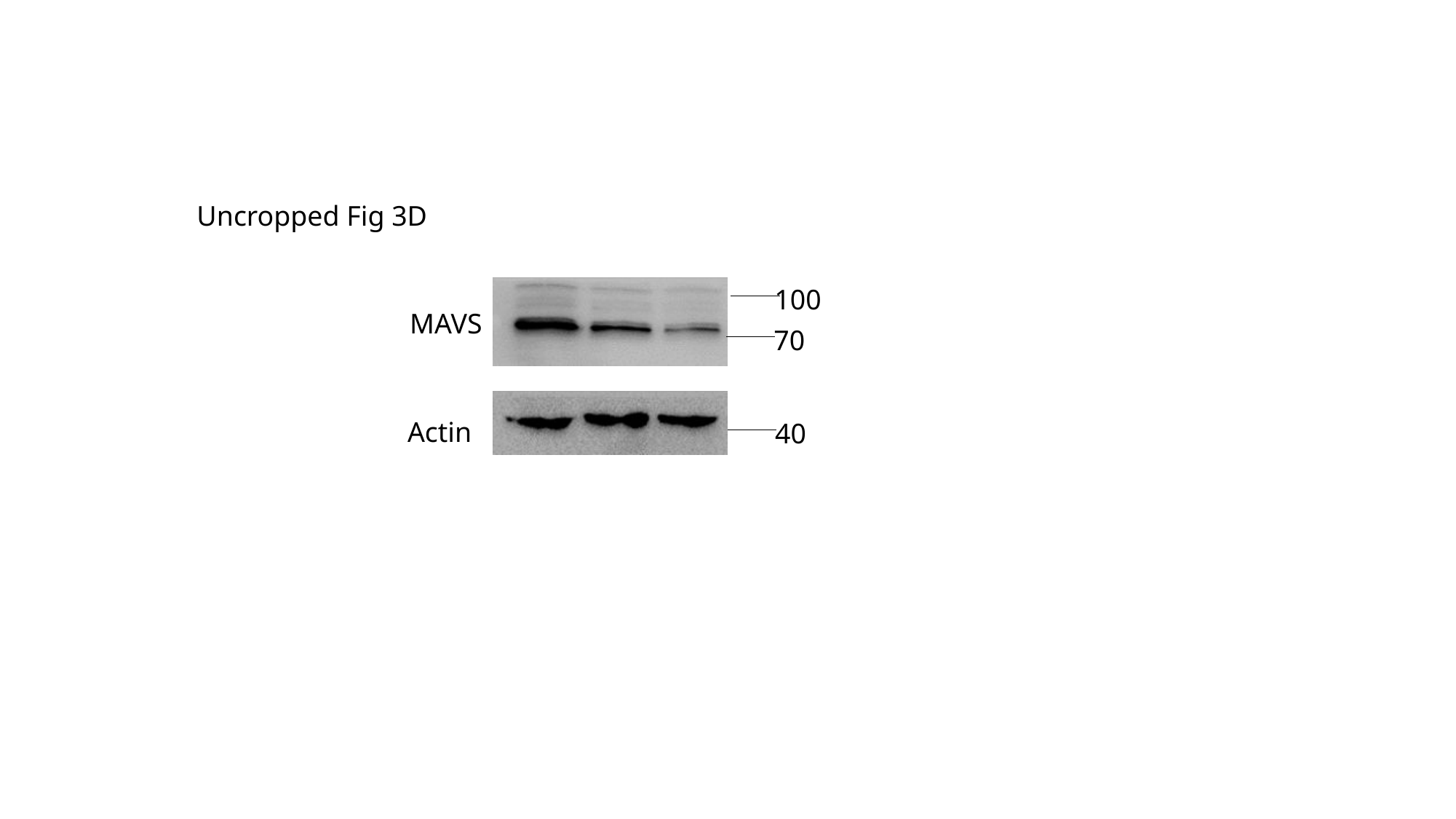

Uncropped Fig 3D
100
MAVS
70
Actin
40

## Slide 8
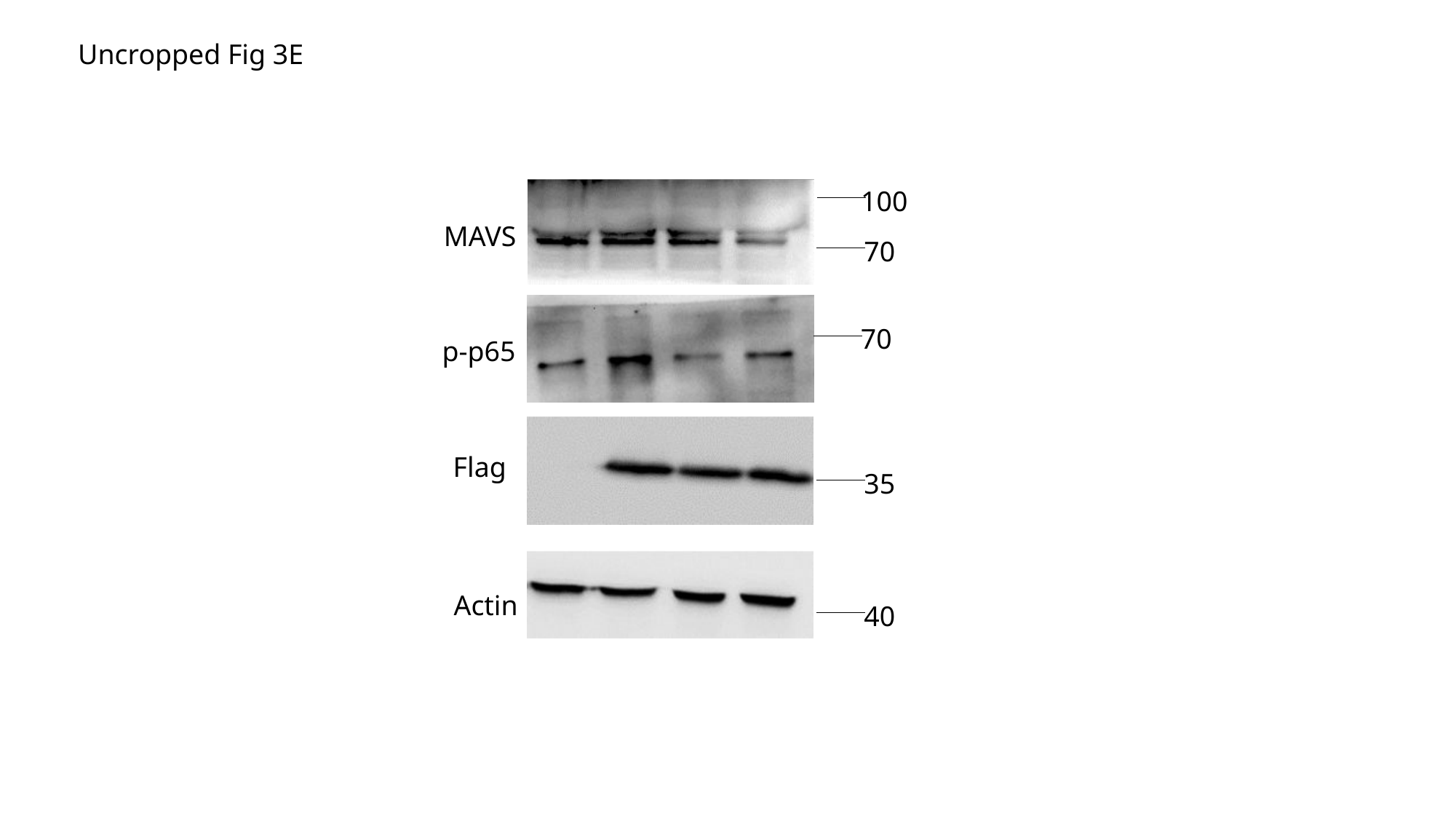

Uncropped Fig 3E
100
MAVS
70
70
p-p65
Flag
35
Actin
40

## Slide 9
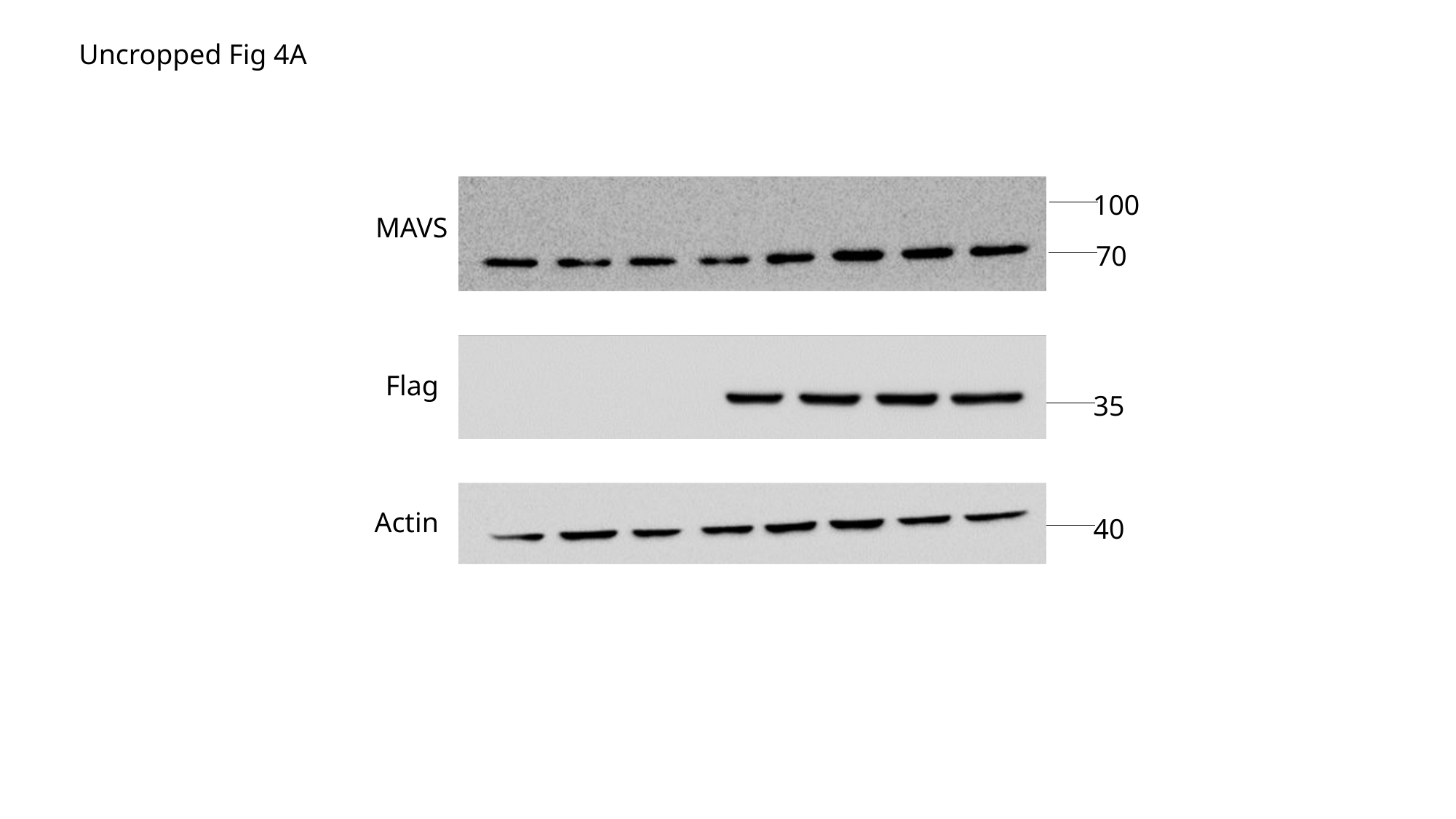

Uncropped Fig 4A
100
MAVS
70
Flag
35
Actin
40

## Slide 10
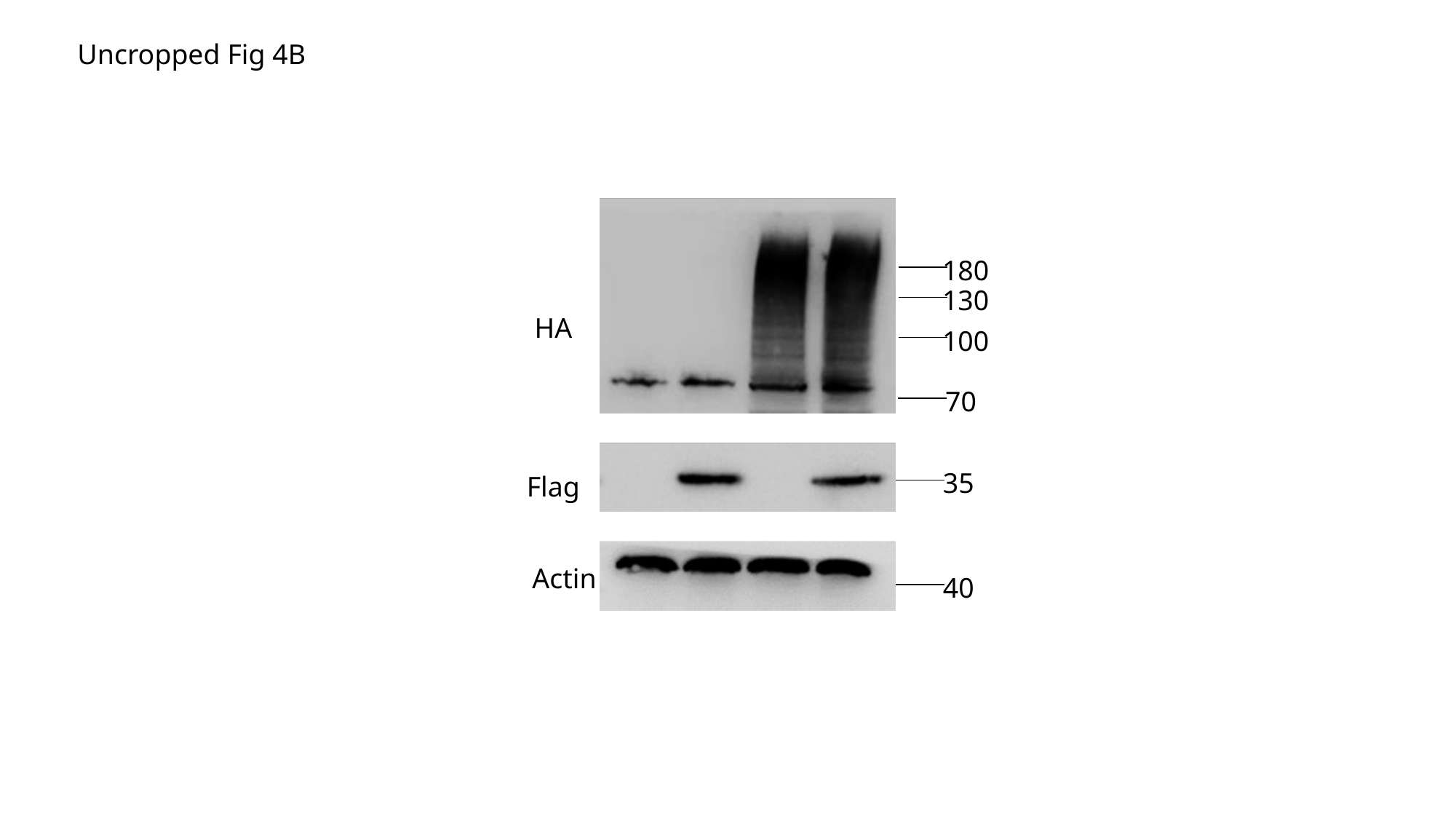

Uncropped Fig 4B
180
130
HA
100
70
35
Flag
Actin
40

## Slide 11
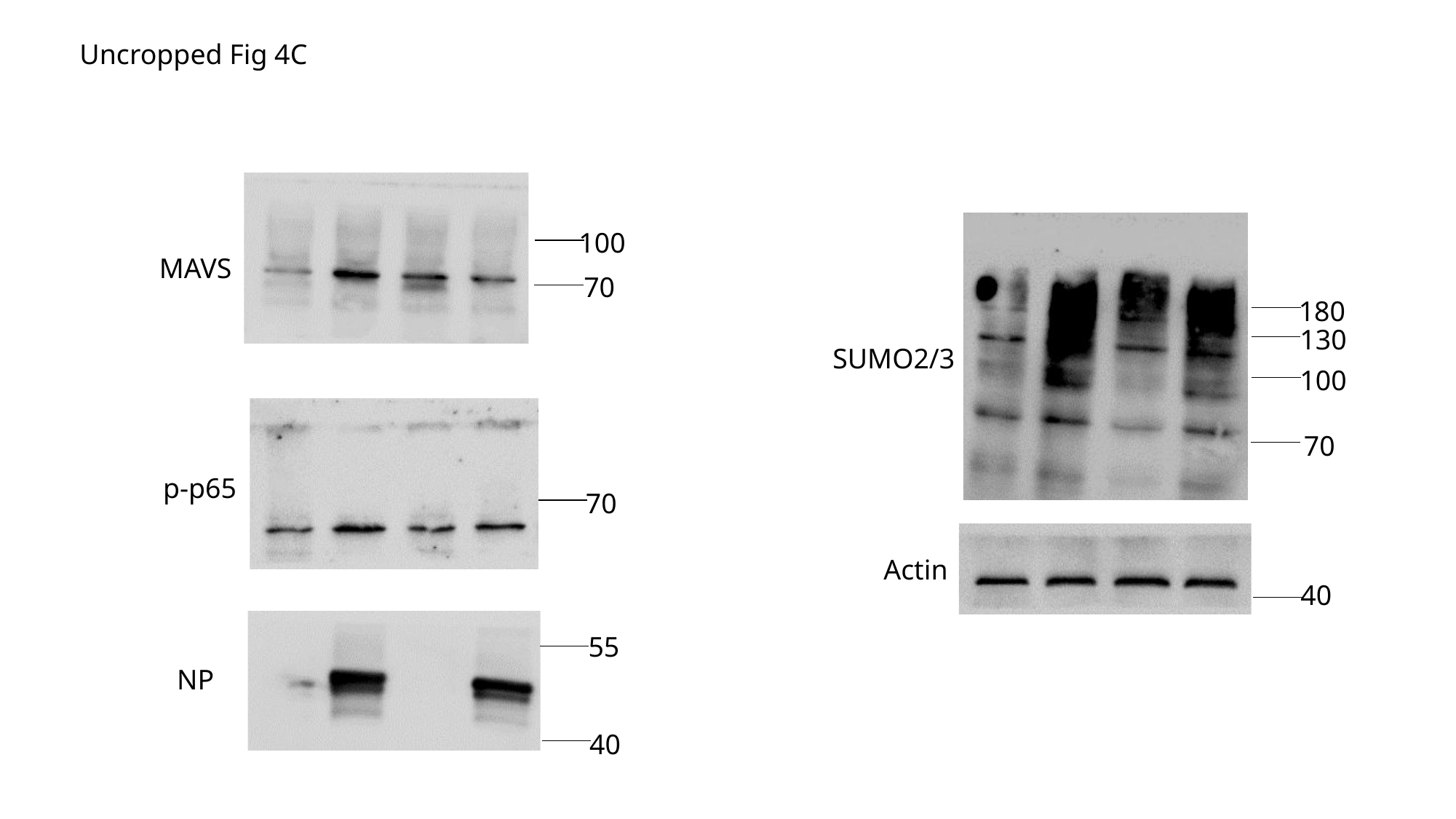

Uncropped Fig 4C
100
MAVS
70
180
130
SUMO2/3
100
70
p-p65
70
Actin
40
55
NP
40

## Slide 12
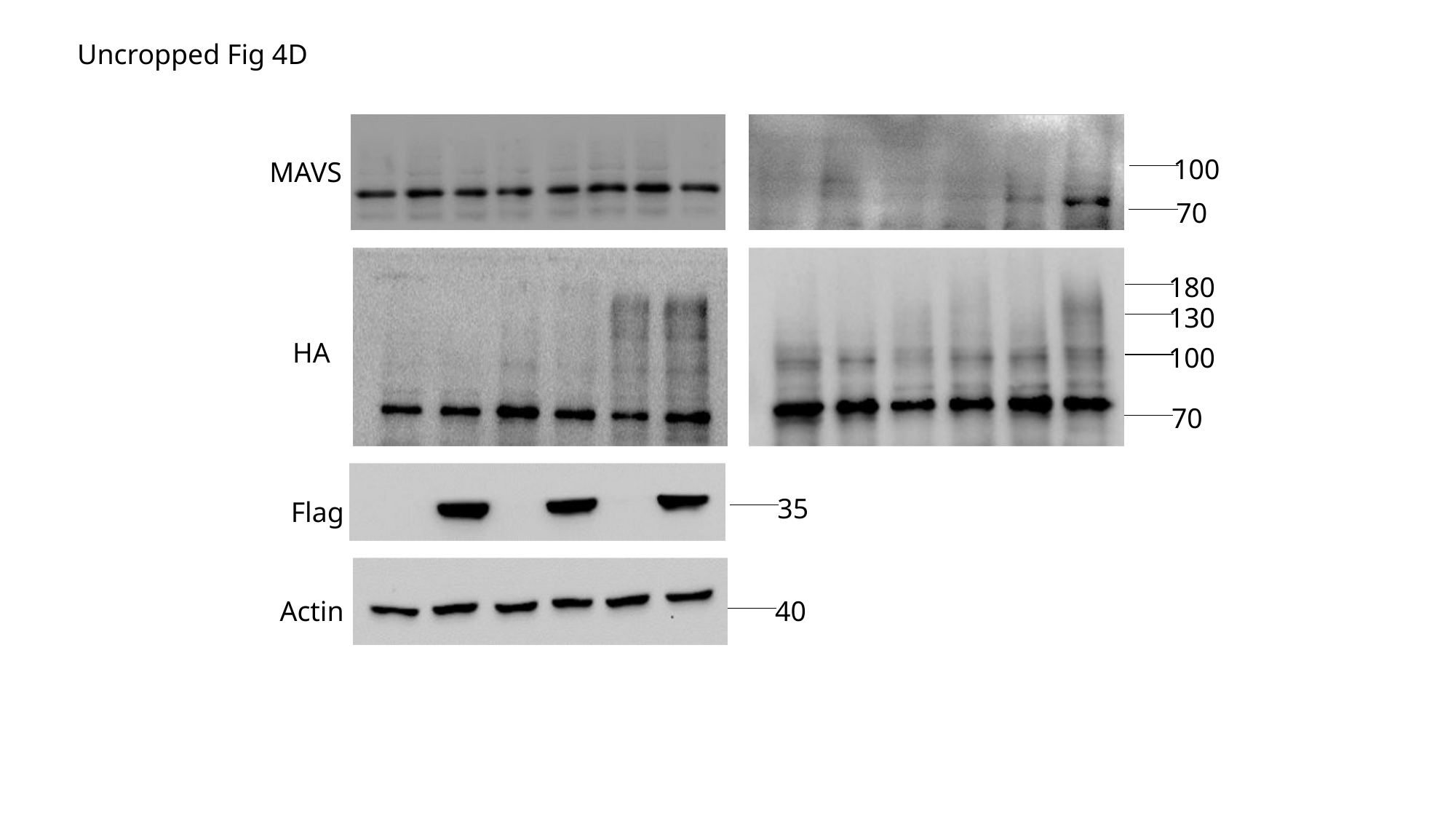

Uncropped Fig 4D
100
MAVS
70
180
130
HA
100
70
35
Flag
Actin
40

## Slide 13
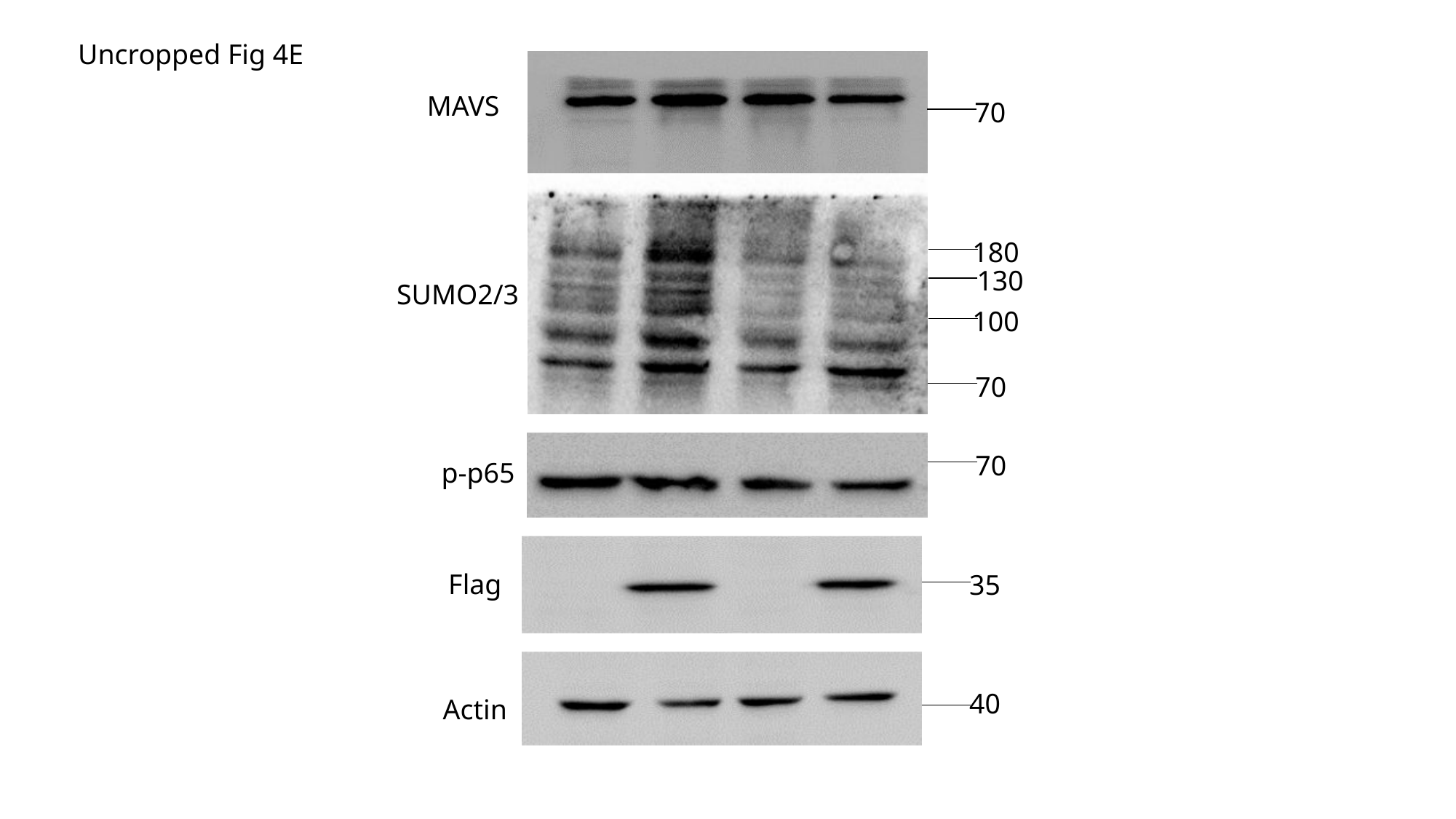

Uncropped Fig 4E
MAVS
70
180
130
SUMO2/3
100
70
70
p-p65
Flag
35
40
Actin
